# In a Protest Nation – Integrative Policy Negotiation Should be a Core Public Health Competency

**DOI:** 10.5334/aogh.3291

**Published:** 2021-04-14

**Authors:** Jeff Lane

**Affiliations:** 1 University of Washington, Department of Global Health, US

## Abstract

The year 2020 was been a year of protest in the United States the likes of which we have not seen in decades. In many ways, America’s history is a history of protest, but its history also shows the power and potential of demonstrations and dialogue to lead to broad coalitions for policy and public health action. The U.S. President’s Emergency Plan for AIDS Relief (PEPFAR) is one example that illustrates the collective power of demonstration and dialogue. To achieve the level of public support needed for meaningful and sustainable responses to major public health challenges, integrative policy negotiation should become a core public health competency. We have developed a series of hypothetical case-based role plays to practice integrative policy negotiation in the context of public health policy advocacy in a hypothetical country called Countryland. These tools are included as appendices and are free to use and adapt. If every public health professional becomes fluent in integrative policy negotiation, maybe we can look back on 2020 as the year that started a new era of pragmatic protest that finally achieves the enduring public health policy changes that we desperately need.

## Viewpoint

More than 50 years ago, Rev. Dr. Martin Luther King Jr. observed: “People fail to get along with each other because they fear each other. They fear each other because they don’t know each other. They don’t know each other because they have not properly communicated with each other [1,2].” In the midst of terrible acts of violence and discrimination against Black people in the United States, Dr. King spoke about the need for demonstration and dialogue across ideological, political, and racial lines as critical steps in successfully advocating for policy reform. Though spoken more than fifty years ago, these words could also be used to describe the current partisan and ideological divide in the United States revealed by COVID-19.

I have been teaching Policy Development and Advocacy for Global Health for the past eight years at a university located in one of the most politically liberal cities in America. Yet, I live in one of the most politically conservative states in America. The ideological divide between liberal and conservative America is palpable as I have traveled between my home state and home university these last few years and is demonstrated by public polling data [3]. The recent politicization of masking, vaccines, and social distancing, and the U.S.’s near-withdrawal from the World Health Organization are just a few examples of how this ideological divide is undermining collaborative local and global public health efforts. Unless this divide can be bridged, I fear that we will continue to struggle to meet our greatest public health challenges, including climate change, racial inequality, universal health care access, and responses to pandemic diseases including (but not limited to) COVID-19.

In the past I have used the advocacy campaign of Dr. King that led to the U.S. Voting Rights Act as a case study in my policy course and found myself reflecting on that case study following the 2020 election. One particular quote from Dr. King has stuck with me these last few weeks: “Every [person] of humane convictions must decide on the protest that best suits [their] convictions, but we must all protest [4].” The year 2020 was a year of protest the likes of which we have not seen in decades. But in many ways, America’s history is a history of protest. American protests and counter-protests go back to the Boston Tea party, and continue through the 13th Amendment, women’s suffrage, civil rights, anti-fascism, anti-communism, anti-McCarthyism, climate change, anti-immigration, immigrant rights, health care reform, the re-envisioned tea party movement, sexual minority rights, anti-racism, and many others. The right to assemble and peacefully protest is even secured as the first amendment to our national Constitution. This history of protest is not surprising, considering that our Constitution was a grand experiment in granting individual rights, but then failed to recognize those rights for so many groups. Americans have been protesting ever since. The 19^th^ century French sociologist Alexis de Tocqueville alluded to this in his early observation that “[t]he greatness of America lies not in being more enlightened than any other nation, but rather in her ability to repair her faults [5].” In many ways, America is a protest nation, but America’s history also shows the power and potential of demonstration and dialogue to lead to broad coalitions for policy and public health action-the kind of action we need now.

The U.S. President’s Emergency Plan for AIDS Relief (PEPFAR) is one example that illustrates the collective power of demonstration and dialogue. In the 1990s and early 2000s, lack of access to lifesaving antiretroviral treatment for people living with HIV was the focus of demonstrations and political advocacy around the world. In the United States, some organizations made the strategic choice to include in their protest strategy efforts to build a broad coalition of politically liberal and conservative members of Congress. This coalition building is perhaps best illustrated by the efforts of U2 rock star Bono’s advocacy to Sen. Jesse Helms (Republican Senator from North Carolina) using Bible verses to frame engaging in the fight against the HIV/AIDS epidemic as an issue of Christian morality [6]. These advocacy campaigns ultimately led to the launch of PEPFAR in 2003 with broad bipartisan support. Over the past 17 years, PEPFAR has contributed more than $90 billion to combat HIV/AIDS around the world. PEPFAR has been the target of criticism at times from the political left and political right, but the core of PEPFAR is built on a consensus platform to provide evidence-based HIV treatment, care, and prevention services. The representatives voting to re-authorize PEPFAR year after year have significant disagreements on many areas of policy. However, due to the breadth and depth of the PEPFAR coalition and its efforts to maintain bipartisanship, the U.S.’s policy commitment to supporting the global fight against HIV/AIDS remains strong and has contributed to saving millions of lives from a pandemic that we are still fighting today.

To achieve the level of public support needed for meaningful and sustainable responses to major public health challenges, we need to make policy negotiation, advocacy, and coalition building core competencies for public health professionals. A negotiation method known as integrative bargaining provides a framework for building and applying these skills. Integrative bargaining is a form of negotiation that emphasizes problem solving, cooperation, joint decision-making, and finding win-win solutions [7]. Integrative bargaining provides a framework for protesting in a way that can lead to broad and powerful coalitions for change. It helps put into practice the advice of Justice Ruth Bader Ginsberg when she said, “Fight for the things that you care about, but do it in a way that will lead others to join you [8].” Fisher and Ury [9] identify seven elements of principled negotiation and integrative bargaining. See ***Table 1*** for a summary of these elements and the implications for public health policy negotiations.

**Table 1:** Seven Elements of Principled Negotiation for Public Health Policy.


ELEMENT OF PRINCIPLED NEGOTIATION	IMPLICATIONS FOR PUBLIC HEALTH POLICY NEGOTIATIONS

Separate People from the Problem	Collegial relationships facilitate cooperation and deal making

Focus on Interests	Seek to understand the goals and concerns of other stakeholders; avoid focusing on specific proposals until you understand the interests motivating other key groups; completing and updating a stakeholder matrix can facilitate this

Generate Options	Think creatively about approaches that address the interests of all key stakeholder groups; think about direct service delivery, regulatory, and finance policy levers

Know Your Best Alternative to a Negotiated Agreement	Analyze the status quo prior to starting a negotiation and critically assess whether a policy proposal would be better than walking away with no change to current policy

Use Objective Criteria	Rely on data and standards (not argument) to inform and guide the negotiation

Follow Through on Commitments	Maintain trust and credibility throughout and after the negotiation; in public health you will often negotiate with the same parties over and over again; you may also need to work with them to implement the policy change

Communicate	Share your perspective and interests to facilitate the negotiation and actively listen to identify the main interests of other key stakeholder groups; frame your policy proposals in a way that speaks to the interests of key stakeholder groups


The people-centered approach of integrative bargaining aligns with many aspects of public health theory. In Social Behavior Change Communication, we seek to understand the interests of specific stakeholder groups and then develop communication strategies and messaging that meets those groups “where they are.” In stigma reduction interventions, we seek to build bridges between community members, health care workers, and stigmatized groups to increase familiarity, empathy, and understanding. As part of our patient-centered approaches, we now use terms such as People Who Inject Drugs, People Who Engage in Transactional Sex, and People with Mental Illness to validate humanity first and avoid labeling the identity of the person in a way that leads to conflict, shame, or defensiveness. These people-centered approaches allow for more effective collaboration, problem solving, and change. Integrative bargaining relies on very similar approaches.

In our course on global health policy and advocacy, we have developed and refined a series of hypothetical case-based role plays over the years to practice integrative policy negotiation in the context of public health policy advocacy. These role plays are set in a hypothetical middle-income country called Countryland. They allow the students to assume the roles of different stakeholder groups within Countryland, such as a governmental official, conservative faith-based organization, patient advocacy group, or even a pharmaceutical company executive. The students role play through different scenarios to practice different aspects of active listening, persuasive communication, and coalition building. We challenge students during these role plays to explore these differing viewpoints and diagnose the interests motivating other stakeholder groups. One scenario is a negotiation on pricing for a patented medication. Another scenario is a stakeholder meeting to discuss harm reduction policies for people who engage in transactional sex. We have also developed policy briefs that analyze a problem of substandard medicines and recommends policy action in Countryland to improve medicine quality. All of these tools are available as Appendices and are free to use, adapt, and build on.

These hypothetical case studies have allowed us to adjust fact patterns and scenarios to highlight key learning objectives and strengthen policy advocacy competencies. For example, instead of tuberculosis, the negotiation role play could be revised to focus on access to a hepatitis C or COVID-19 vaccine. These role plays allow students to assume roles that they may not otherwise have the opportunity to play, and even assume the role of a group for which they have strong pre-determined negative feelings. We have even successfully implemented some of these role plays using video conferencing with students located around the world. This same approach could be replicated with students located in more politically liberal or conservative areas to add diversity of opinion or experience to the course. Student evaluations over the years have consistently cited these integrative bargaining role plays as key contributors to their learning.

As Dr. King said, “There is some good in the worst of us and some evil in the best of us. When we discover this, we are less prone to hate our enemies [10].” We are all humans whose opinions, behaviors, and perspectives are influenced by our experiences and biases (myself included), but history has shown that those opinions and perspectives can also evolve through new experiences and dialogue. As we enter 2021, perhaps we can reflect on our history of protest and the lessons it could offer on our plans for protest in 2021. The principled and pragmatic approach of integrative policy negotiation could be one method for a protest strategy that seeks to build a vision of a future that is supported by broad and powerful coalitions that are less susceptible to cyclical partisan fluctuations (like the coalition supporting PEPFAR). If every public health professional becomes fluent in integrative policy negotiation, maybe we can look back on 2020 as the year that started a new era of pragmatic protest-one that demonstrates and demands change, but also principled cross-cultural dialogue and coalition building that finally achieves the enduring public health policy changes that we desperately need.

## Additional Files

The additional files for this article can be found as follows:

10.5334/aogh.3291.s1 Appendices. The appendices include facilitator guides and small group instructions for two stakeholder negotiation role plays regarding: (1) harm reduction for people in Countryland who engage in transactional sex; and (2) access to a patented medicine to address a disease outbreak in Countryland. The appendices also include an example policy brief from Countryland that describes a specific public health problem regarding prevalence of substandard and falsified medicines in Countryland and an evaluation of potential policy interventions to address this problem.
