# Supplementary material for: In a Protest Nation – Integrative Policy Negotiation Should be a Core Public Health Competency
Source: Ann Glob Health. 2021 Apr 14;87(1):38. doi: 10.5334/aogh.3291 (PMC8051155; doi:10.5334/aogh.3291)
Supplement: Appendices. — The appendices include facilitator guides and small group instructions for two stakeholder negotiation role plays regarding: (1) harm reduction for people in Countryland who engage in transactional sex; and (2) access to a patented medicine to address a disease outbreak in Countryland. The appendices also include an example policy brief from Countryland that describes a specific public health problem regarding prevalence of substandard and falsified medicines in Countryland and an evaluation of potential policy interventions to address this problem. [file agh-87-1-3291-s1.zip › agh-87-1-3291-s1/3291-11591-1-SP.pdf]

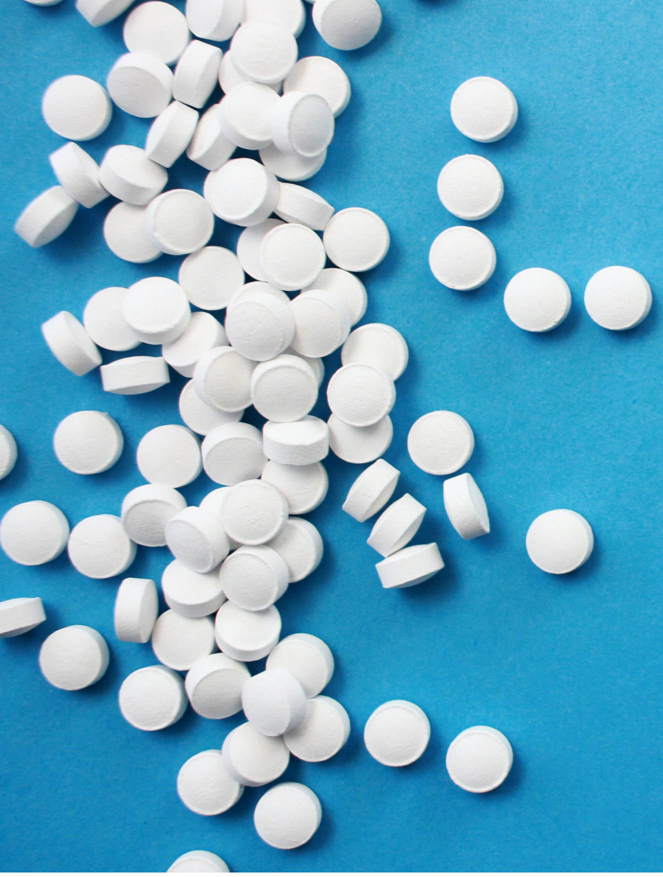

Improving Access to Quality Antimalarial Medicines for Countryland Children

Results of a Multi-Criteria Policy Analysis

Executive Summary

Malaria is one of the most significant public health threats facing children in Countryland today, and substandard and falsified antimalarial medications are undermining the government's control efforts. Data indicate that perhaps one-third of antimalarial medications may be substandard or falsified. Substandard and falsified antimalarial medication can lead to prolonged and more severe disease, drug resistance, and even death. A substantial percentage of medications in Countryland are purchased from private pharmacies and drug shops, especially in rural areas. Therefore, it is critical that the quality of antimalarial medication sold in private pharmacies and drug shops in rural areas be a focus of any policy aimed at combating substandard and falsified antimalarial medication.

We evaluated three policy options intended to reduce the prevalence of substandard and falsified antimalarial medication at private drug shops and pharmacies: (1) expanding the number of public pharmacies, especially in rural areas; (2) hiring additional drug regulatory authority inspectors to increase frequency of private pharmacy and drug shop inspections; and (3) establishing a medication voucher program that would allow public sector patients to purchase medicines at accredited private pharmacies that have undergone more rigorous inspection. We evaluated all three options against three criteria: implementation feasibility, cost, and equity.

We identified strengths and weaknesses with all three options, but on balance, we recommend prioritizing **hiring additional pharmacy inspectors to enforce existing regulations at private pharmacies and drug shops**. Options 1 and 3 could also be considered as part of a comprehensive antimalarial quality control program.

WHO Definitions of Substandard and Falsified Medical Products (2017)

Substandard medications are medical products authorized for medical use that fail to meet quality standards or specifications set out by a governing body, such as the WHO or a country's Ministry of Health.

Falsified products deliberately misrepresent their true identity, composition, or source

This hypothetical role play is part of a collection of tools and case studies set in a hypothetical middle-income country called Countryland.

Background

Malaria continues to be a major public health threat in Countryland, especially for children. In 2019, Countryland had an under-five malaria prevalence rate of 20%.(1) In 2019, malaria was also responsible for 35% of all pediatric hospitalizations in Countryland.(1) It has been estimated that up to one-third of antimalarial medication in the region may substandard or falsified.(2) Substandard and falsified antimalarial medication can lead to treatment failure, drug resistance, and even death.(1) A substantial percentage of medications in Countryland are purchased from private pharmacies and drug shops. For example, 2017 study found that approximately 60% of parents of children with malaria in rural Eastern Countryland initially purchased antimalarial medication from private pharmacies or drug shops. (3)

20%

Malaria Prevalence for Children Under 5 Years of Age

National Drug Authority (NDA) Act is the main law governing medicines regulation in Countryland. Regulations adopted in 2016 under the NDA Act establish licensing requirements for private pharmacies. In 2017, Countryland launched a National Medicines Policy (NMP) and National Medicines Sector Strategic Plan. The NDA is the primary government agency responsible for ensuring the quality of medicines in Countryland. The NDA's Department of Inspections and Enforcement, is responsible for monitoring quality through retail chains, including wholesalers, drug shops, and pharmacies. A 2017 analysis conducted by the NDA and international organizations found that the NDA has limited human resources undermining its ability to enforce quality and safety regulations at private pharmacies and drug shops.(3)

Policy Options

We evaluated three policy options to address the problem of substandard and falsified antimalarial medication in private sector drug shops and pharmacies.

Option 1 - Expand the number of public pharmacies operated by the Central Medical Store (CMS), especially in rural areas. Expanding the network of public pharmacies will allow patients to access medicines procured through the CMS system, instead of having to rely on private pharmacies, which are challenging to regulate.

Option 2 - Hire additional NDA drug inspectors to increase frequency of private pharmacy and drug shop inspections.

Option 3 - Establish a medicine voucher program where patients living in areas without a public pharmacy can use the voucher to purchase medicines from private pharmacies that are accredited by the NDA as selling quality medicines. To be accredited, the private pharmacy would need to participate in more stringent inspections and quality oversight by the NDA. This would allow the NDA to focus its inspections on a smaller number of private pharmacies and create an incentive for private pharmacies to seek accreditation.

Analysis Criteria

We evaluated these three policy options against the following three criteria:

Implementation Feasibility – This criterion assessed the complexity of implementing this intervention and the amount of time and effort that would be required to prepare for successful implementation

Cost – This criterion assessed the financial and material costs of the intervention in the short and long-term

Equity – This criterion assessed the potential effect of this intervention on populations with the greatest needs

60% **Percentage of Parents Who Purchase Antimalarials from Private Pharmacies or Drug Shops**

Analysis of Option 1

Implementation Feasibility - Expanding the number of public pharmacies would be complex from an implementation standpoint. The sites would need to be identified and new pharmacy buildings or retail spaces would need to be constructed or renovated. Medicine supply chain systems would also need to be strengthened and modified to serve these new locations. Additional pharmacists may need to be hired and possibly trained and it may be difficult to identify trained pharmacists to work in some rural areas. While there may be a number of implementation challenges, a strength of this approach is that the government already operates many public pharmacies and the delivery models for existing pharmacies could be replicated for new pharmacies. [Score: 2]

Cost - Increasing the number of public pharmacies would be very costly. Each new pharmacy may require construction and would require additional human resources and supplies. Task shifting may be an option for reducing human resources costs, but even with increased efficiency, significantly increasing the number of public pharmacies would be a costly response to this problem. The estimated budget impact would depend on the number of new pharmacies and their locations. [Score: 2]

Equity - Increasing the number of public pharmacies in areas that are currently underserved by the existing pharmacy network would make this intervention responsive to the needs of patients in greatest need. Close oversight of site selection and developing a site selection methodology that prioritizes population need would be important to ensure that new pharmacies would be located in areas with greatest need. [Score: 4]

Total Score - Option 1 (8/15)

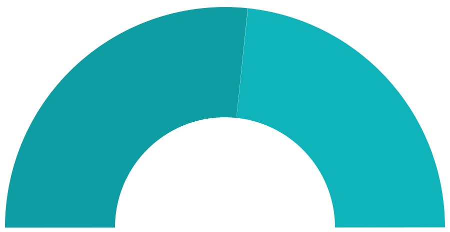

Analysis of Option 2

Implementation Feasibility - Hiring additional drug inspectors at the NDA would be fairly simple from an implementation standpoint. The DRA has an existing cadre of inspectors. The NDA may face barriers in recruiting and retaining these new inspectors if salaries are not adequate and it is unclear whether there is an adequate supply of qualified candidates in the country, but presumably inspectors could be trained by existing NDA inspectors. Overall, the implementation approach for this intervention is clear with only minimal risks. [Score: 5]

Cost - Hiring additional pharmacy inspectors would be relatively costly. New inspectors would require salary and benefits in addition to the current NDA human resources budget. The budget impact of this intervention would depend on the total number of new inspectors hired and the retention rates. The number of new inspectors hired could be adjusted based on the amount of additional resources identified. Costs could potentially be offset by increasing licensing rates or licensing fees for private sector pharmacies and drug shops. [Score: 3]

Equity - Hiring additional drug inspectors to inspect private pharmacies would not necessarily have a greater benefit to populations with the greatest need. If inspections were focused on private pharmacies serving populations with limited access to public pharmacies, the equity score for this intervention could be higher. [Score: 2]

Total Score - Option 2 (10/15)

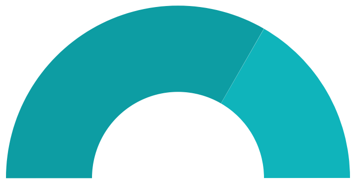

Analysis of Option 3

Implementation Feasibility - Developing a medicine voucher program for accredited private pharmacies would be complex from an implementation standpoint. The eligibility criteria for patients and accreditation standards for private pharmacies would need to be developed and an administrative oversight structure would need to be established. There would be a range of implementation risks including the potential for fraud, potential lack of interest from private pharmacies to participate, and the challenges with implementing an administrative structure to operate the program. For this reason, implementation of this intervention would be complex and would face significant implementation risks. [Score: 1]

Cost - Cost of developing a private pharmacy voucher program is difficult to assess at this point because a number of variables remain unknown. Budget impact would depend on who is eligible to receive vouchers, the price for medicines procured through the system, and the administrative structure required to oversee the system. For this reason, cost is given a relatively low score because of the number of unknowns surrounding this intervention and the potential for very high cost. [Score: 1]

Equity - Developing a voucher program would have a number of strengths from the perspective of improving health equity. The program could be designed to target individuals with the greatest need and existing private pharmacies serving populations with greatest need could be targeted for accreditation. A weakness of this approach is that it could divert public funding from the Central Medical Store and public pharmacy system, which is critical to serving the needs of low-income families in Countryland. [Score: 4]

Total Score - Option 3 (6/15)

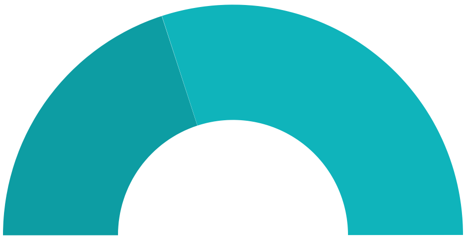

Total Score - Option 1 (8/15)

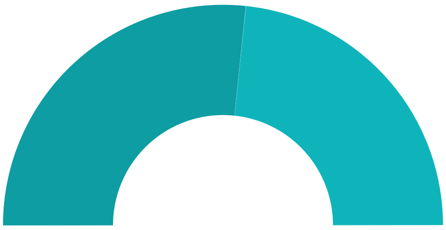

Total Score - Option 2 (10/15)

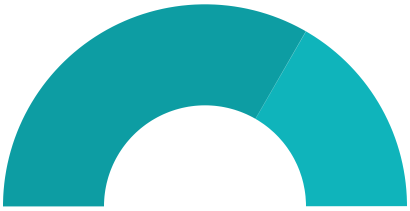

Total Score - Option 3 (6/15)

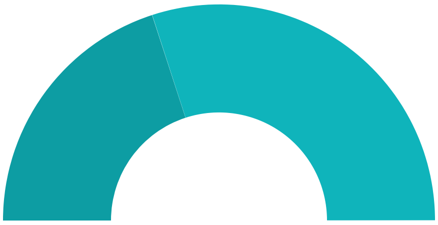

Recommendation

We identified strengths and weaknesses with all three options, but on balance, we recommend prioritizing the option of the **hiring additional pharmacy inspectors to enforce existing regulations at private pharmacies and drug shops**. Options 1 and 3 could also be considered as part of a comprehensive antimalarial quality control program.

Key References

1. Peer Reviewed Journal - Analysis of Countryland Medicine Quality (2019)

2. Global Report on Substandard and Falsified Medicines (2010)

3. Peer Reviewed Journal - Countryland Substantard Medicine Analysis (2017)
