# Supplementary material for: In a Protest Nation – Integrative Policy Negotiation Should be a Core Public Health Competency
Source: Ann Glob Health. 2021 Apr 14;87(1):38. doi: 10.5334/aogh.3291 (PMC8051155; doi:10.5334/aogh.3291)
Supplement: Appendices. — The appendices include facilitator guides and small group instructions for two stakeholder negotiation role plays regarding: (1) harm reduction for people in Countryland who engage in transactional sex; and (2) access to a patented medicine to address a disease outbreak in Countryland. The appendices also include an example policy brief from Countryland that describes a specific public health problem regarding prevalence of substandard and falsified medicines in Countryland and an evaluation of potential policy interventions to address this problem. [file agh-87-1-3291-s1.zip › agh-87-1-3291-s1/3291-11592-1-SP.docx]

**Facilitator Guide**

**Commercial Sex Work in Countryland**

**Introduction:** This role-play is designed to allow students to apply the methods of integrated bargaining and principled negotiation regarding a policy proposal to adopt harm reduction interventions for commercial sex work.

**In-Class Role Play Structure:** The students should be divided into 5 small groups and assigned one of the hypothetical stakeholder groups (the Countryland Police Force, Tourism and Trade Association of Countryland, the Countryland Coalition of Faith-Based Organizations, Countryland Public Health Association, and the Coalition for Commercial Sex Worker Rights). Each group should be provided approximately 15-30 minutes to prepare in a small group for the role play and answer the questions included on the confidential role play instructions. The groups should then be brought back together in plenary. The instructor will play the role as the Prime Minister of Countryland and open the meeting by summarizing the situation and asking for input from the stakeholder groups. The Prime Minister should also state how long the meeting will last (e.g., 45 minutes) so groups can plan accordingly. The Prime Minister then calls on each stakeholder group and ask them to present their views on the interventions or approaches that should be included in the proposal. The Prime Minister can emphasize that the she/he would like consensus from the group on the interventions to be included in the policy. The Prime Minister can then ask questions of specific groups to try to highlight differences of opinion to allow groups to respond to other groups in the room and to provide groups opportunities to reframe their positions to speak to the interests of other groups. The Prime Minister could write on the white board/chalk board specific interventions to gauge which groups are supportive of specific types of interventions.

**In-Class Time Required:** 1-1.5 hours (15-30 minutes of small group preparation; 30-45 minutes of role play; 10-20 minutes of debrief)

**Debrief:** During the debrief, the instructor can ask the students the following questions:

- Do you think you were able to identify the interests of other stakeholder groups? What helped you identify the interests of other groups?
- Who was the primary audience for messaging?
- Which other stakeholder groups did you target and why?

**Competencies:**

- Apply integrative bargaining and principled negotiation methods to build a coalition to address a global health problem
- Identify and describe interests of diverse stakeholder groups
- Design an advocacy strategy to influence positions of key stakeholder groups

**Small Group Instructions**

**Tourism and Trade Association of Countryland**

The Prime Minister of Countryland is receiving international pressure to address a growing HIV/AIDS epidemic in the country and epidemiology data indicates that transactional sex work is a driver of the epidemic. The Prime Minister’s party made the eradication of child prostitution a campaign issue during recent elections, but the Prime Minister’s position on adult commercial sex work is unclear. All forms of commercial sex work are technically illegal in Countryland, but enforcement has been lax. International sex tourism has contributed to the country’s economy in the past, but some groups are now concerned that the commercial sex trade is undermining the country’s reputation as a family-friendly tourism destination. The Prime Minister has convened a stakeholder consultation meeting to receive input on a potential policy change that would emphasize harm reduction interventions for adults who engage in transactional sex. The invited stakeholder groups are: the Countryland Police Force, Tourism and Trade Association of Countryland, the Countryland Coalition of Faith-Based Organizations, Countryland Public Health Association, and the Coalition for Commercial Sex Worker Rights. The invitation for the stakeholder meeting include the below content from a Lancet article that the Prime Minster read on this topic:

Sex work is an extremely dangerous profession. The use of harm-reduction principles can help to safeguard sex workers’ lives in the same way that drug users have benefited from drug-use harm reduction. Sex workers are exposed to serious harms: drug use, disease, violence, discrimination, debt, criminalisation, and exploitation (child prostitution, trafficking for sex work, and exploitation of migrants). Successful and promising harm-reduction strategies are available: education, empowerment, prevention, care, occupational health and safety, decriminalisation of sex workers, and human-rights-based approaches. Successful interventions include peer education, training in condom-negotiating skills, safety tips for street-based sex workers, male and female condoms, the prevention-care synergy, occupational health and safety guidelines for brothels, self-help organisations, and community-based child protection networks. Straightforward and achievable steps are available to improve the day-to-day lives of sex workers while they continue to work. Conceptualising and debating sex-work harm reduction as a new paradigm can hasten this process. *[M Rekart, Sex-Work Harm Reduction, Lancet 2005; 366: 2123–34.]*

Your group represents the **Tourism and Trade Association of Countryland**. The Prime Minister has a close relationship with the Tourism and Trade Association (which includes hotel owners, airlines, restaurateurs, and tour agencies), and the Prime Minister recognizes tourism's importance in the national economy. The trade group is interested in reducing HIV/AIDS in Countryland, because fear that stigma around HIV/AIDS could keep tourists away. It wants something to be done regarding child prostitution, because it is bad for the country’s international reputation, but association members fear that increased restrictions on all forms of commercial sex work and increased attention on the issue will be bad for business.

**Discussion Questions** - Discuss and answer these questions before the role play begins:

1. What are three interests of this stakeholder group regarding this issue?
2. What are three interests of the Prime Minister that your group will try to appeal to?
3. What are three major considerations and/or specific interventions that you think the Prime Minister should include in this policy?

**Small Group Instructions**

**Coalition for Commercial Sex Worker Rights**

The Prime Minister of Countryland is receiving international pressure to address a growing HIV/AIDS epidemic in the country and epidemiology data indicates that transactional sex work is a driver of the epidemic. The Prime Minister’s party made the eradication of child prostitution a campaign issue during recent elections, but the Prime Minister’s position on adult commercial sex work is unclear. All forms of commercial sex work are technically illegal in Countryland, but enforcement has been lax. International sex tourism has contributed to the country’s economy in the past, but some groups are now concerned that the commercial sex trade is undermining the country’s reputation as a family-friendly tourism destination. The Prime Minister has convened a stakeholder consultation meeting to receive input on a potential policy change that would emphasize harm reduction interventions for adults who engage in transactional sex. The invited stakeholder groups are: the Countryland Police Force, Tourism and Trade Association of Countryland, the Countryland Coalition of Faith-Based Organizations, Countryland Public Health Association, and the Coalition for Commercial Sex Worker Rights. The invitation for the stakeholder meeting include the below content from a Lancet article that the Prime Minster read on this topic:

Sex work is an extremely dangerous profession. The use of harm-reduction principles can help to safeguard sex workers’ lives in the same way that drug users have benefited from drug-use harm reduction. Sex workers are exposed to serious harms: drug use, disease, violence, discrimination, debt, criminalisation, and exploitation (child prostitution, trafficking for sex work, and exploitation of migrants). Successful and promising harm-reduction strategies are available: education, empowerment, prevention, care, occupational health and safety, decriminalisation of sex workers, and human-rights-based approaches. Successful interventions include peer education, training in condom-negotiating skills, safety tips for street-based sex workers, male and female condoms, the prevention-care synergy, occupational health and safety guidelines for brothels, self-help organisations, and community-based child protection networks. Straightforward and achievable steps are available to improve the day-to-day lives of sex workers while they continue to work. Conceptualising and debating sex-work harm reduction as a new paradigm can hasten this process. *[M Rekart, Sex-Work Harm Reduction, Lancet 2005; 366: 2123–34.]*

Your group represents the **Coalition for Commercial Sex Worker Rights (CCSWR)**. CCSWR is a civil society organization that advocates for protecting the rights of commercial sex workers. CCSWR is officially opposed to child prostitution. Some staffers within CCSWR have advocated for legalizing and regulating adult consensual commercial sex work, but CCSWR has not taken an official position on the legalization of adult commercial sex work. CCSWR provides job training and harm reduction support to adult and child commercial sex workers. CCSWR thinks that it is unfair for commercial sex workers coerced into working in the commercial sex trade to be prosecuted as criminals. Some staffers at CCSWR are also concerned that a crackdown on adult consensual commercial sex work will drive the industry further underground.

**Discussion Questions** - Discuss and answer these questions before the role play begins:

1. What are three interests of this stakeholder group regarding this issue?
2. What are three interests of the Prime Minister that your group will try to appeal to?
3. What are three major considerations and/or specific interventions that you think the Prime Minister should include in this policy?

**Small Group Instructions**

**Coalition of Faith-based Organizations**

The Prime Minister of Countryland is receiving international pressure to address a growing HIV/AIDS epidemic in the country and epidemiology data indicates that transactional sex work is a driver of the epidemic. The Prime Minister’s party made the eradication of child prostitution a campaign issue during recent elections, but the Prime Minister’s position on adult commercial sex work is unclear. All forms of commercial sex work are technically illegal in Countryland, but enforcement has been lax. International sex tourism has contributed to the country’s economy in the past, but some groups are now concerned that the commercial sex trade is undermining the country’s reputation as a family-friendly tourism destination. The Prime Minister has convened a stakeholder consultation meeting to receive input on a potential policy change that would emphasize harm reduction interventions for adults who engage in transactional sex. The invited stakeholder groups are: the Countryland Police Force, Tourism and Trade Association of Countryland, the Countryland Coalition of Faith-Based Organizations, Countryland Public Health Association, and the Coalition for Commercial Sex Worker Rights. The invitation for the stakeholder meeting include the below content from a Lancet article that the Prime Minster read on this topic:

Sex work is an extremely dangerous profession. The use of harm-reduction principles can help to safeguard sex workers’ lives in the same way that drug users have benefited from drug-use harm reduction. Sex workers are exposed to serious harms: drug use, disease, violence, discrimination, debt, criminalisation, and exploitation (child prostitution, trafficking for sex work, and exploitation of migrants). Successful and promising harm-reduction strategies are available: education, empowerment, prevention, care, occupational health and safety, decriminalisation of sex workers, and human-rights-based approaches. Successful interventions include peer education, training in condom-negotiating skills, safety tips for street-based sex workers, male and female condoms, the prevention-care synergy, occupational health and safety guidelines for brothels, self-help organisations, and community-based child protection networks. Straightforward and achievable steps are available to improve the day-to-day lives of sex workers while they continue to work. Conceptualising and debating sex-work harm reduction as a new paradigm can hasten this process. *[M Rekart, Sex-Work Harm Reduction, Lancet 2005; 366: 2123–34.]*

You represent the **Coalition of Faith-Based Organizations (CFBO).** The Prime Minister received significant support from CFBO in the last election, and CFBO now appears to have significant influence over the Prime Minister’s decisions. CFBO is firmly against all forms of prostitution (child and adult) and opposes any efforts that appear to tolerate what CFBO considers to be immoral and scandalous behavior. CFBO supports programs to help women escape prostitution, like job training, but is very skeptical of “harm reduction” strategies that do not require women to give up commercial sex work. CFBO believes that international sex tourism must be rooted out of the country completely regardless of the economic effects. To CFBO, prostitution is an issue of moral principle.

**Discussion Questions** - Discuss and answer these questions before the role play begins:

1. What are three interests of this stakeholder group regarding this issue?
2. What are three interests of the Prime Minister that your group will try to appeal to?
3. What are three major considerations and/or specific interventions that you think the Prime Minister should include in this policy?

**Small Group Instructions**

**Countryland Police Force**

The Prime Minister of Countryland is receiving international pressure to address a growing HIV/AIDS epidemic in the country and epidemiology data indicates that transactional sex work is a driver of the epidemic. The Prime Minister’s party made the eradication of child prostitution a campaign issue during recent elections, but the Prime Minister’s position on adult commercial sex work is unclear. All forms of commercial sex work are technically illegal in Countryland, but enforcement has been lax. International sex tourism has contributed to the country’s economy in the past, but some groups are now concerned that the commercial sex trade is undermining the country’s reputation as a family-friendly tourism destination. The Prime Minister has convened a stakeholder consultation meeting to receive input on a potential policy change that would emphasize harm reduction interventions for adults who engage in transactional sex. The invited stakeholder groups are: the Countryland Police Force, Tourism and Trade Association of Countryland, the Countryland Coalition of Faith-Based Organizations, Countryland Public Health Association, and the Coalition for Commercial Sex Worker Rights. The invitation for the stakeholder meeting include the below content from a Lancet article that the Prime Minster read on this topic:

Sex work is an extremely dangerous profession. The use of harm-reduction principles can help to safeguard sex workers’ lives in the same way that drug users have benefited from drug-use harm reduction. Sex workers are exposed to serious harms: drug use, disease, violence, discrimination, debt, criminalisation, and exploitation (child prostitution, trafficking for sex work, and exploitation of migrants). Successful and promising harm-reduction strategies are available: education, empowerment, prevention, care, occupational health and safety, decriminalisation of sex workers, and human-rights-based approaches. Successful interventions include peer education, training in condom-negotiating skills, safety tips for street-based sex workers, male and female condoms, the prevention-care synergy, occupational health and safety guidelines for brothels, self-help organisations, and community-based child protection networks. Straightforward and achievable steps are available to improve the day-to-day lives of sex workers while they continue to work. Conceptualising and debating sex-work harm reduction as a new paradigm can hasten this process. *[M Rekart, Sex-Work Harm Reduction, Lancet 2005; 366: 2123–34.]*

You represent the **Countryland Police Force**. The police have traditionally had a close relationship with the ruling political party. It is widely believed that some police officials supplement their low salaries by extorting money from brothels and other sex establishments. The police force has been embarrassed in the past by media reports that police regularly tip off brothel owners before a raid. Some brothels raided in the past have actually included the term "police costs” on their accounting records. Some police officials have also taken a strong public stand against corruption, including corruption linked with the commercial sex industry. Police are aware that some groups may be advocating to legalize adult commercial sex work, but they are concerned how to implement an age requirement in Countryland where fake IDs are widely available.

**Discussion Questions** - Discuss and answer these questions before the role play begins:

1. What are three interests of this stakeholder group regarding this issue?
2. What are three interests of the Prime Minister that your group will try to appeal to?
3. What are three major considerations and/or specific interventions that you think the Prime Minister should include in this policy?

**Small Group Instructions**

**Countryland Public Health Association**

The Prime Minister of Countryland is receiving international pressure to address a growing HIV/AIDS epidemic in the country and epidemiology data indicates that transactional sex work is a driver of the epidemic. The Prime Minister’s party made the eradication of child prostitution a campaign issue during recent elections, but the Prime Minister’s position on adult commercial sex work is unclear. All forms of commercial sex work are technically illegal in Countryland, but enforcement has been lax. International sex tourism has contributed to the country’s economy in the past, but some groups are now concerned that the commercial sex trade is undermining the country’s reputation as a family-friendly tourism destination. The Prime Minister has convened a stakeholder consultation meeting to receive input on a potential policy change that would emphasize harm reduction interventions for adults who engage in transactional sex. The invited stakeholder groups are: the Countryland Police Force, Tourism and Trade Association of Countryland, the Countryland Coalition of Faith-Based Organizations, Countryland Public Health Association, and the Coalition for Commercial Sex Worker Rights. The invitation for the stakeholder meeting include the below content from a Lancet article that the Prime Minster read on this topic:

Sex work is an extremely dangerous profession. The use of harm-reduction principles can help to safeguard sex workers’ lives in the same way that drug users have benefited from drug-use harm reduction. Sex workers are exposed to serious harms: drug use, disease, violence, discrimination, debt, criminalisation, and exploitation (child prostitution, trafficking for sex work, and exploitation of migrants). Successful and promising harm-reduction strategies are available: education, empowerment, prevention, care, occupational health and safety, decriminalisation of sex workers, and human-rights-based approaches. Successful interventions include peer education, training in condom-negotiating skills, safety tips for street-based sex workers, male and female condoms, the prevention-care synergy, occupational health and safety guidelines for brothels, self-help organisations, and community-based child protection networks. Straightforward and achievable steps are available to improve the day-to-day lives of sex workers while they continue to work. Conceptualising and debating sex-work harm reduction as a new paradigm can hasten this process. *[M Rekart, Sex-Work Harm Reduction, Lancet 2005; 366: 2123–34.]*

You represent the **Countryland Public Health Association**. The Public Health Association has made HIV/AIDS one of its top priorities for political advocacy. Studies have found that HIV prevalence among female sex workers in some parts of the country is over 40%. The Public Health Association has advocated in the past for adopting harm reduction strategies for adult and child commercial sex workers and sees this conference as an opportunity to shine a light on the public health crisis facing commercial sex workers in Countryland.

**Discussion Questions** - Discuss and answer these questions before the role play begins:

1. What are three interests of this stakeholder group regarding this issue?
2. What are three interests of the Prime Minister that your group will try to appeal to?
3. What are three major considerations and/or specific interventions that you think the Prime Minister should include in this policy?
