# Supplementary material for: In a Protest Nation – Integrative Policy Negotiation Should be a Core Public Health Competency
Source: Ann Glob Health. 2021 Apr 14;87(1):38. doi: 10.5334/aogh.3291 (PMC8051155; doi:10.5334/aogh.3291)
Supplement: Appendices. — The appendices include facilitator guides and small group instructions for two stakeholder negotiation role plays regarding: (1) harm reduction for people in Countryland who engage in transactional sex; and (2) access to a patented medicine to address a disease outbreak in Countryland. The appendices also include an example policy brief from Countryland that describes a specific public health problem regarding prevalence of substandard and falsified medicines in Countryland and an evaluation of potential policy interventions to address this problem. [file agh-87-1-3291-s1.zip › agh-87-1-3291-s1/3291-11593-1-SP.docx]

**Facilitator Guide**

**Introduction:** This role-play is designed to allow students to apply the methods of integrated bargaining and principled negotiation to build coalitions to address a global health problem.

**Preparation (approx. 60 minutes):** Students should read the common role-play background reading and the following paper by Alfredson and Cungu on negotiation theory with a focus on the section on integrative bargaining (pp. 18-25): *Alfredson, T., Cungu, A. (2008). Negotiation Theory and Practice: A Review of the Literature. Food and Agriculture Organization of the United Nations, FAO*.

**In-Class Role Play Structure:** The students should be divided into 5 small groups and assigned one of the hypothetical stakeholder groups. Each group should be provided approximately 30 minutes to prepare in a small group for the negotiation and answer the questions included on the confidential negotiating instructions. The groups should then be brought back together in plenary. The instructor will play the role as the mediator of the negotiation and open the negotiation by summarizing the situation and encouraging the parties to try to come together to find an agreement on a way forward. The mediator can provide an overview of the negotiating process that will consist of multiple rounds of each group being allowed to make statements with short (e.g., 5 minute) caucus breaks. During these caucus breaks small groups can discuss among themselves or meet with other stakeholder groups for side conversations to try to build coalitions or find common ground. The mediator can help facilitate the process by writing the interests and/or proposals described by each stakeholder group on a white board and update those throughout the negotiation. As the negotiation proceeds, the mediator may need to call on the MOH and PharmaCo groups more than other groups to encourage those two groups to come to agreement. Other stakeholder groups are key stakeholders, but ultimately the two groups with decision-making authority are the MOH and PharmaCo. Setting a specific amount of time for the negotiation (approximately 60-90 minutes) may be important to help groups come together on an agreement. MOH and PharmaCo may or may not be able to come to an agreement during that amount of time.

**In-Class Time Required:** 2.5-3 hours (30-45 minutes of small group preparation; 60-90 minutes of role play; 15-30 minutes of debrief) [this exercise can be separated into multiple class sessions]

**Debrief:** During the debrief, the instructor can ask the students the following questions:

- Do you think you were able to identify the interests of other stakeholder groups? What helped you identify the interests of other groups?
- What steps (e.g., arguments, questions, standards) helped advance the negotiation?
- What was it like to participate in a negotiation where you did not have decision-making authority? How did that affect your approach?

**Competencies:**

- Apply integrative bargaining and principled negotiation methods to build a coalition to address a global health problem
- Identify and describe interests of diverse stakeholder groups
- Design an advocacy strategy to influence positions of key stakeholder groups

**Background Reading for All Small Groups**

Countryland has been hit hard by a new strain of Extensively Drug-Resistant Tuberculosis (“XDR-TB”). The new strain is extremely contagious and has a higher than normal fatality rate. The Government of Countryland estimates that approximately 5,000 Countrylanders have contracted this new strain of XDR-TB and urgently need treatment.

Last year, PharmaCo, a for-profit pharmaceutical company based in the United States, announced a new antibiotic (Expensifin) that is effective at treating this new strain of TB. Expensifin must be taken on a daily basis for 12-months to be effective. Shortly before the announcement, PharmaCo filed patent applications for Expensifin in the US, European Union, Canada, India, Brazil, South Africa and every other country with a substantial pharmaceutical manufacturing industry (but not Countryland). PharmaCo has set the price for Expensifin at $10,000 for a twelve month course of treatment. Countryland does not have a substantial pharmaceutical manufacturing sector and the Ministry of Health (MOH) has estimated that it may take up to five years to establish a local manufacturing site to produce essential medicines like Expensifin.

The MOH of Countryland would like to offer Expensifin at its existing public DOTS facilities. However, the cost to the MOH for meeting the annual need for Expensifin based on PharmaCo’s published price would be $50 million USD per year ($10,000/course of treatment x 5,000 patients = $50 million). That cost does not include other costs associated with a TB program, including human resources for health, DOTS management, facilities, laboratory, or supply chain costs. For context, last year the MOH spent a total of 500 million USD on health services.

Generia, a generic medicine manufacturer located in India, has publicly estimated that it could manufacture and sell Expensifin for $200 per person for a 12-month course of treatment. There are elections in Countryland in one year, and access to Expensifin is becoming an increasingly hot button political issue that the minority party in Countryland is starting to use against the majority party and the Prime Minister.

Your organization has been invited to a meeting between various stakeholders to negotiate a strategy for making Expensifin available in Countryland. The following groups have been invited to the meeting: Countryland MOH, PharmaCo, World Health Organization, Pharmaceutical Company Association (PCA), and the Coalition for Patient Rights (CPR). A mediator will be present to help facilitate the negotiation.

**Confidential Negotiating Instructions**

**Ministry of Health of Countryland**

The Ministry of Health of Countryland (MOH) has publicly committed to make Expensifin available at public DOTS clinics, but it cannot purchase the drug at the published price. The MOH can only afford up to $800 per person. If the price is over $800 per person, the MOH will need to establish a wait list. The MOH’s budget is already set for the year, and therefore any money spent on Expensifin will redirect funds away from other high priority program areas. So the lower the price, the better.

The MOH is facing an enormous amount of political pressure from the Coalition for Patient Rights (CPR) to make Expensifin available as soon as possible. CPR is a coalition of nonprofits from across Countryland with chapters in other countries and was a strong supporter of the current Prime Minister.

Because of the urgent need, the MOH does not see establishing a public pharmaceutical manufacturing facility in Countryland to be a viable option for Expensifin, because it would take 5 years to build that capacity. The Government of Countryland has discussed the possibility of issuing a compulsory license to a foreign manufacturer in India called Generia. Generia has publicly announced that it could manufacture and sell a generic version of Expensifin at $200 per person, but only if Generia is issued a compulsory license for Expensifin or if PharmaCo voluntarily licenses Expensifin to Generia directly or through the Medicines Patent Pool. However, Generia has privately told the MOH that it could take one year or more for Generia to produce its first dose.

Some in the Government of Countryland are also concerned that the governments of high income countries might retaliate against Countryland in international trade deal negotiations if Countryland issues a compulsory license to Generia.

**During the preparation time, your group should discuss and complete the following questions to guide your negotiation:**

1. At least three 3 interests that will guide your negotiating strategy:
2. 2-3 potential interests of PharmaCo that you will try to appeal to:
3. Your starting price offer to PharmaCo:
4. Your Best Alternative to a Negotiated Agreement (i.e., your best option if you walk away from the negotiation with no deal):

**Confidential Negotiating Instructions**

**PharmaCo**

PharmaCo thinks Expensifin could be blockbuster antibiotic for a range of bacterial infections, including certain types of infections that are common in high income countries. For this reason, PharmaCo is keenly interested in protecting its patent monopoly on this drug. PharmaCo has also identified the middle and high income segments of South Africa and India as prime markets for Expensifin. PharmaCo has an Access Initiative that can make medicines available free of charge to people who qualify as very poor. PharmaCo is open to setting up such an office in Countryland to review applications for free Expensifin as long as PharmaCo retains the right to set the income criteria.

PharmaCo’s CEO has personally told your team that the PharmaCo’s Board of Directors will be monitoring the negotiations carefully and wants to ensure that PharmaCo does not come off as insensitive to the public health needs of people in Countryland. However, the Board of Directors also wants to make sure that PharmaCo comes off as a fierce protector of its intellectual property rights. The Board is concerned that if PharmaCo is rolled during these negotiations that other countries, especially South Africa and India, and PharmaCo’s competitors may take a more adversarial approach with PharmaCo regarding intellectual property rights on a range of other medicines. The Board of Directors sees Countryland issuing a compulsory license to a foreign manufacturer to be the worst possible outcome.

The Board has authorized your team to open the negotiations with an offer to reduce the price down to $5,000 per person, and has authorized you to ultimately negotiate the price down to $750 per person, *but only as a last resort to avoid a compulsory license*. Alternatively, PhrmaCo would agree to license Expensifin to the Medicines Patent Pool, if other middle-income countries (except Countryland) are excluded from the license. Granting a voluntary license to the Medicine Patent Pool The CEO and Board of Directors will need to endorse whatever you agree to, so if your negotiating strategy does not follow the instructions set out by the CEO and Board of Directors, they will reject it (and your team will likely be fired).

**During the preparation time, your group should discuss and complete the following questions to guide your negotiation:**

1. At least three 3 interests of PharmaCo that will guide your negotiating strategy:
2. 3 potential interests of the Countryland MOH that you will try to appeal to:
3. Your starting price offer to PharmaCo:
4. Your Best Alternative to a Negotiated Agreement (i.e., your best option if you walk away from the negotiation with no deal):

**Confidential Negotiating Instructions**

**World Health Organization**

The World Health Organization (WHO) is the directing and coordinating authority for health within the United Nations system. It is responsible for providing leadership on global health matters, shaping the health research agenda, setting norms and standards, articulating evidence-based policy options, providing technical support to countries and monitoring and assessing health trends. The WHO Director-General has asked your team to participate in the Countryland Expensifin negotiations. Because Countryland is at the center of the current epidemic, WHO thinks it is critical that Expensifin be available immediately in Countryland. The Director-General is very concerned that this strain of MDR-TB will become a global pandemic if Expensifin is not made widely available in the very near future in all countries. As a result, the Director-General would also like your team to see if you can get PharmaCo to make Expensifin available in all middle-income and low-income countries at low cost.

The Director-General is intrigued by the idea of using the Medicines Patent Pool for this purpose, but would be open to other voluntary licenses. The WHO is a United Nations agency, so the WHO generally prefers negotiated resolutions to medicine intellectual property disputes as opposed to compulsory licenses.

**During the preparation time, your group should discuss and complete the following questions to guide your negotiation:**

1. At least three 3 interests that will guide your negotiating strategy:
2. 2-3 potential interests of the Countryland MOH and PharmaCo that you will try to appeal to:
3. Your starting price offer to PharmaCo:
4. Your Best Alternative to a Negotiated Agreement (i.e., your best option if you walk away from the negotiation with no deal):

**Confidential Negotiating Instructions**

**Coalition for Patient Rights**

The Coalition for Patient Rights (CPR) is a coalition of nonprofit organizations from across Countryland. CPR is very politically active and supported the Countryland Prime Minister in the most recent elections.

CPR has been the loudest advocate for increasing access to Expensifin in Countryland and one of the Prime Minister’s loudest critics on the issue. CPR wants immediate and universal access to Expensifin through public clinics, even if the government has to pay high prices for access to the drug. In fact, CPR would like to see the Countryland Government dramatically increase its spending on health generally. Ideally, the Countryland MOH would get a low price on Expensifin so it could direct health spending to other CPR priority areas.

CPR has sister chapters in other middle-income and low-income countries, which are just as interested in ensuring that their countries have universal access to Expensifin. Thus, CPR’s President has asked your team to see if you can use these negotiations to get PharmaCo to agree to a broader agreement to make Expensifin available at low cost in all middle-income and low-income countries.

**During the preparation time, your group should discuss and complete the following questions to guide your negotiation:**

1. At least three 3 interests that will guide your negotiating strategy:
2. 2-3 potential interests of the Countryland MOH and PharmaCo that you will try to appeal to:
3. Your starting price offer to PharmaCo:
4. Your Best Alternative to a Negotiated Agreement (i.e., your best option if you walk away from the negotiation with no deal):

**Confidential Negotiating Instructions**

**Pharmaceutical Company Association**

The Pharmaceutical Company Association (PCA) is a trade association representing pharmaceutical research and manufacturing companies. PCA’s self-proclaimed mission is to advocate for public policies that encourage discovery of important new medicines for patients. PCA’s goals are to achieve the following: (1) broad patient access to safe and effective medicines through a free market, without price controls; (2) strong intellectual property incentives; and (3) transparent, effective regulation and a free flow of information to patients.

PCA has been invited to this meeting because other pharmaceutical companies have been extremely vocal in the media regarding the Expensifin controversy and PharmaCo has asked PCA to participate. The President of PCA has asked your team to take a strong position against dramatically lowering prices or issuing a voluntary license to a generic manufacturer. The PCA President thinks that because Countryland’s is a middle-income country, it can afford to pay middle-income country prices for Expensifin. Moreover, he believes that it is in PCA members’ interests to control prices and offer drugs free of charge to very poor patients only when the company decides that the patient meets certain financial need criteria, such as PharmaCo’s Access Initiative.

PCA’s President wants your team to remind all of the parties that without PharmaCo’s investment in clinical trials for Expensifin, the world would still have no treatment for this strain of TB. It is likely that TB will continue to evolve and become resistant to other drugs, including Expensifin, and continued research and development into new antibiotics will be critical to ensuring that new anti-TB drugs continue to come to market. The President of PCA thinks it is critical that middle-income countries like Countryland pay higher prices for drugs like Expensifin so that the market for R&D into TB drugs continues to be attractive.

**During the preparation time, your group should discuss and complete the following questions to guide your negotiation:**

1. At least three 3 interests that will guide your negotiating strategy:
2. 2-3 potential interests of the Countryland MOH and PharmaCo that you will try to appeal to:
3. Your starting price offer to PharmaCo:
4. Your Best Alternative to a Negotiated Agreement (i.e., your best option if you walk away from the negotiation with no deal):
